# Polychotomization of continuous variables in regression models based on the overall *C *index

**DOI:** 10.1186/1472-6947-6-41

**Published:** 2006-12-14

**Authors:** Harukazu Tsuruta, Leon Bax

**Affiliations:** 1 Department of Medical Informatics, School of Allied Health Sciences, Kitasato University, Sagamihara, Kanagawa, 228-8555, Japan; 2 Department of Medical Informatics, Graduate School of Medical Sciences, Kitasato University, Sagamihara, Kanagawa, 228-8555, Japan

## Abstract

**Background:**

When developing multivariable regression models for diagnosis or prognosis, continuous independent variables can be categorized to make a prediction table instead of a prediction formula. Although many methods have been proposed to dichotomize prognostic variables, to date there has been no integrated method for polychotomization. The latter is necessary when dichotomization results in too much loss of information or when central values refer to normal states and more dispersed values refer to less preferable states, a situation that is not unusual in medical settings (e.g. body temperature, blood pressure). The goal of our study was to develop a theoretical and practical method for polychotomization.

**Methods:**

We used the overall discrimination index *C*, introduced by Harrel, as a measure of the predictive ability of an independent regressor variable and derived a method for polychotomization mathematically. Since the naïve application of our method, like some existing methods, gives rise to positive bias, we developed a parametric method that minimizes this bias and assessed its performance by the use of Monte Carlo simulation.

**Results:**

The overall *C *is closely related to the area under the ROC curve and the produced di(poly)chotomized variable's predictive performance is comparable to the original continuous variable. The simulation shows that the parametric method is essentially unbiased for both the estimates of performance and the cutoff points. Application of our method to the predictor variables of a previous study on rhabdomyolysis shows that it can be used to make probability profile tables that are applicable to the diagnosis or prognosis of individual patient status.

**Conclusion:**

We propose a polychotomization (including dichotomization) method for independent continuous variables in regression models based on the overall discrimination index *C *and clarified its meaning mathematically. To avoid positive bias in application, we have proposed and evaluated a parametric method. The proposed method for polychotomizing continuous regressor variables performed well and can be used to create probability profile tables.

## Background

In modern diagnostic and descriptive prognostic research, regression models are often used to model an illness-related outcome based on a number of independent regressor variables, also referred to as diagnostic indicators or prognostic predictors [1]. Such regressor variables can be categorical or numerical. From the vantage point of applicability in a clinical setting, categorization (often dichotomization) of continuous independent variables can be useful. Obtaining a prediction at the bedside without computer is easier with a prediction table based on categorized variables than with a prediction formula. Even if calculation is not problematic, table presentation of the risks has the practical advantages that (1) repeated use of the table will give physicians an intuitive feel for the disease risk, and (2) even if the value of one or two of the prognostic variables is not available, physicians can obtain a probability range corresponding to the patient's risk by referring to the most extreme cases in the table.

Depending on the setting, several different approaches have been proposed for dichotomization. One popular method is to find a cutoff point to discriminate whether a patient belongs to a normal group or a disease group based on the observed value of a predictive factor. This type of discriminant function analysis was first developed by R.A. Fisher [2] in 1930's. The Mahalanobis distance [3] can be used to find the optimal cutoff point if the variable distributes normally.

Another solution, sometimes used in clinical chemistry, is to find a cutoff point that maximizes the sum of sensitivity (SE) and specificity (SP) [4,5]. There are different versions of this approach where one can maximize the weighted sum of SE and SP, or maximize the SE while fixing SP to an acceptable value [6,7]. Cantor claimed that these methods have been used in many published articles without giving a theoretical foundation or scientific justification [8].

Yet another straightforward and popular method is to select a classification that maximizes a measure of difference between the two groups, such as the *p*-value of a chi square statistic [9,10]. This method, sometimes called the minimum *p*-value approach, has been described and used for the prognosis of cancers [11,12]. Several authors have pointed out that the naïve selection used in this method overestimates the significance of the predictor or indicator's relationship to the dependent variable because of multiple testing, and several adjustment methods of the observed *p*-values have been proposed [9-18].

Besides using the data at hand to come to a dichotomization of continuous variables, it is also possible to use profit (benefit) or loss (cost) information. In that case, the optical cutoff point is defined so as to maximize the expected utility. Metz showed that the optimal point is the spot on the ROC curve at which the slope is (*L*/*B*)(1-*p*)/*p*, where *B *is the net benefit of treating diseased individuals, *L *the net loss of treating non-diseased individuals, and *p *the prevalence of the disease under study [19]. Nevertheless, Cantor et al., in a review of studies in the medical literature that referred to "ROC" and "cutoff", found that only a few articles included a *L*/*B *ratio in the analysis for determining an optimal cutoff point [8].

The above methods all concern dichotomization. However, when central values refer to normal states and dispersed values to diseased states, two (or more) cutoff points are necessary to discriminate these states. Consequently, one is inevitably faced with the challenge of polychotomization. Unfortunately, methods for polychotomization are less developed. Although Kristjansson et al. [20] described a method for choosing optimal cutoff points in a screening test with a continuous score to divide people into a number of disease categories, their method is not applicable to polychotomization of regressor variables in regression models; their criterion loses its meaning in this setting.

The major goal of our study is to develop a theoretical and practical method for polychotomization. We propose a novel approach for independent continuous variables in regression models based on the overall discrimination index *C *introduced by Harrel et al. [21,22]. We will show that this index is closely related to the area under the ROC curve for the original continuous variable and that the resulting categorized variables have predictive properties comparable to the original continuous variable. However, the naïve search of the maximum *C *index gives rise to positive bias, not unlike the minimum *p*-value approach [9-18] or the method of maximizing the sum of the sensitivity and specificity [4,5]. We therefore propose a parametric version in which the estimates of the predictive performance and cutoff points are both essentially unbiased. We evaluate this method and present means and standard deviations of predictive performance and cutoff point estimates for typical cases via Monte Carlo simulation. Finally, we provide a simple application example with a predictive regression model for rhabdomyolysis and show how our method can be used to create a probability profile table.

## Methods

### The categorization criterion

We assume there is an existing predictive model based on patients that belong to either a normal group or a diseased group and that the distribution of the relevant independent continuous variable *X *is known or that we have observations on it. Our goal is to find a method of optimal polychotomization for this continuous variable with a minimum loss of predictive ability. This involves making the number of possible patient's profiles finite, and replacing the regression formula with a table of the risk probabilities for all patient profiles. Different from most previously developed approaches we have no a priori intention to categorize the variable into two classes and we assume that it might be necessary to compare categorizations to three or more classes.

For this discussion we need a measure to evaluate the predictive power of a predictive variable. Our choice for a measure of predictive power is the overall discrimination index *C *[21-24], or the 'pair consistency probability', as we like to call it. This measure refers to the probability that the relative position of single normal-disease pair values is consistent with the relative position of their values of central tendency.

Without losing generality, we assume that the central value of the distribution of the random variable *X *in the group of healthy cases is smaller than the central value in the group of diseased cases. Next we take a sample *x*_*i*[*h*] _from the healthy group and another sample *x*_*i*[*d*] _from the diseased group randomly. Then the pair (*x*_*i*[*h*]_, *x*_*i*[*d*]_) is considered *consistent *if *x*_*i*[*h*] _<*x*_*i*[*d*]_, *tied *if *x*_*i*[*h*] _= x_*i*[*d*]_, and *inconsistent *if *x*_*i*[*h*] _> *x*_*i*[*d*] _and the pair consistency probability *C *is defined as:

C=pcon+12ptied,     (1)
 MathType@MTEF@5@5@+=feaafiart1ev1aaatCvAUfKttLearuWrP9MDH5MBPbIqV92AaeXatLxBI9gBaebbnrfifHhDYfgasaacH8akY=wiFfYdH8Gipec8Eeeu0xXdbba9frFj0=OqFfea0dXdd9vqai=hGuQ8kuc9pgc9s8qqaq=dirpe0xb9q8qiLsFr0=vr0=vr0dc8meaabaqaciaacaGaaeqabaqabeGadaaakeaacqWGdbWqcqGH9aqpcqWGWbaCdaWgaaWcbaGaem4yamMaem4Ba8MaemOBa4gabeaakiabgUcaRmaalaaabaGaeGymaedabaGaeGOmaidaaiabdchaWnaaBaaaleaacqWG0baDcqWGPbqAcqWGLbqzcqWGKbazaeqaaOGaeiilaWIaaCzcaiaaxMaadaqadaqaaiabigdaXaGaayjkaiaawMcaaaaa@42FB@

where *p*_*con *_and *p*_*tied *_denote the probabilities that the pair is consistent and tied respectively.

Next, if we let *f*_*h *_represent the probability density function (PDF) of *X *in the healthy group and *f*_*d *_represent the PDF of *X *in the diseased group, and let *z *represent a cutoff point for dichotomization, then the true positive fraction *Tp *and false positive fraction *Fp *are defined by

Tp=∫z∞fd(x)dx and Fp=∫z∞fh(x)dx.
 MathType@MTEF@5@5@+=feaafiart1ev1aaatCvAUfKttLearuWrP9MDH5MBPbIqV92AaeXatLxBI9gBaebbnrfifHhDYfgasaacH8akY=wiFfYdH8Gipec8Eeeu0xXdbba9frFj0=OqFfea0dXdd9vqai=hGuQ8kuc9pgc9s8qqaq=dirpe0xb9q8qiLsFr0=vr0=vr0dc8meaabaqaciaacaGaaeqabaqabeGadaaakeaacqWGubavcqWGWbaCcqGH9aqpdaWdXaqaaiabdAgaMnaaBaaaleaacqWGKbazaeqaaOGaeiikaGIaemiEaGNaeiykaKcaleaacqWG6bGEaeaacqGHEisPa0Gaey4kIipakiabdsgaKjabdIha4jabbccaGiabbggaHjabb6gaUjabbsgaKjabbccaGiabdAeagjabdchaWjabg2da9maapedabaGaemOzay2aaSbaaSqaaiabdIgaObqabaGccqGGOaakcqWG4baEcqGGPaqkaSqaaiabdQha6bqaaiabg6HiLcqdcqGHRiI8aOGaemizaqMaemiEaGNaeiOla4caaa@5629@

In the case that the variable is continuous, as *z *increases, *Tp *and *Fp *both decrease continuously. The ROC curve [19,25] can be depicted as the trace of points (*Fp *, *Tp *). Green and Swets [25] demonstrated that

C=∫−∞∞P(x[h]=z)⋅P(x[d]>z)dz=∫−∞∞fh(z)⋅[∫z∞fd(x)dx]dz=∫01Tp(z)dFp(z).
 MathType@MTEF@5@5@+=feaafiart1ev1aaatCvAUfKttLearuWrP9MDH5MBPbIqV92AaeXatLxBI9gBaebbnrfifHhDYfgasaacH8akY=wiFfYdH8Gipec8Eeeu0xXdbba9frFj0=OqFfea0dXdd9vqai=hGuQ8kuc9pgc9s8qqaq=dirpe0xb9q8qiLsFr0=vr0=vr0dc8meaabaqaciaacaGaaeqabaqabeGadaaakqaaeeqaaiabdoeadjabg2da9maapedabaGaemiuaaLaeiikaGIaemiEaG3aaSbaaSqaaiabcUfaBjabdIgaOjabc2faDbqabaGccqGH9aqpcqWG6bGEcqGGPaqkcqGHflY1cqWGqbaucqGGOaakcqWG4baEdaWgaaWcbaGaei4waSLaemizaqMaeiyxa0fabeaakiabg6da+iabdQha6jabcMcaPaWcbaGaeyOeI0IaeyOhIukabaGaeyOhIukaniabgUIiYdGccqWGKbazcqWG6bGEaeaacqGH9aqpdaWdXaqaaiabdAgaMnaaBaaaleaacqWGObaAaeqaaOGaeiikaGIaemOEaONaeiykaKIaeyyXICTaei4waS1aa8qmaeaacqWGMbGzdaWgaaWcbaGaemizaqgabeaakiabcIcaOiabdIha4jabcMcaPiabdsgaKjabdIha4jabc2faDjabdsgaKjabdQha6bWcbaGaemOEaOhabaGaeyOhIukaniabgUIiYdaaleaacqGHsislcqGHEisPaeaacqGHEisPa0Gaey4kIipaaOqaaiabg2da9maapedabaGaemivaqLaemiCaaNaeiikaGIaemOEaONaeiykaKIaemizaqMaemOrayKaemiCaaNaeiikaGIaemOEaONaeiykaKIaeiOla4caleaacqaIWaamaeaacqaIXaqma0Gaey4kIipaaaaa@84FF@

This means that the pair consistency probability is equivalent to the area under the ROC curve for continuous variables. We will demonstrate that this relation also holds for polychotomized variables, and that the pair consistency probability *C *is a good measure to compare the predictive ability with the original continuous variable.

### Optimal cutoff point for dichotomization

First, we discuss our method for dichotomization in which a continuous independent variable in a predictive model is categorized to one of two classes by a cutoff point. If we denote the value of the cutoff point *z *and assume that *X *is continuous in both the healthy and the diseased groups, that is, P(*x*_[*h*] _= *z*) = 0 and P(*x*_ [*d*] _= *z*) = 0, the results of random pair sampling are classified into the following four cases:

*x*_[*h*] _<*z *and *x*_[*d*] _<*z*,     *tied*

*x*_[*h*] _<*z *and *x*_[*d*] _> *z*,     *consistent*

*x*_[*h*] _> *z *and *x*_[*d*] _<*z*,     *inconsistent*

*x*_[*h*] _> *z *and *x*_[*d*] _> *z*,     *tied*.

Let *α *denote the probability that *x*_[*h*] _is greater than *z*, and *β *denote the probability that *x*_[*d*] _is less than *z*. Assuming that the central value of the distribution of the random variable *X *in the group of healthy cases is smaller than the central value in the group of diseased cases, we have

α=∫z∞fh(x)dx=Fp and β=∫−∞zfd(x)dx=1−Tp.     (2)
 MathType@MTEF@5@5@+=feaafiart1ev1aaatCvAUfKttLearuWrP9MDH5MBPbIqV92AaeXatLxBI9gBaebbnrfifHhDYfgasaacH8akY=wiFfYdH8Gipec8Eeeu0xXdbba9frFj0=OqFfea0dXdd9vqai=hGuQ8kuc9pgc9s8qqaq=dirpe0xb9q8qiLsFr0=vr0=vr0dc8meaabaqaciaacaGaaeqabaqabeGadaaakeaaiiGacqWFXoqycqGH9aqpdaWdXaqaaiabdAgaMnaaBaaaleaacqWGObaAaeqaaOGaeiikaGIaemiEaGNaeiykaKIaemizaqMaemiEaGNaeyypa0JaemOrayKaemiCaaNaeeiiaaIaeeyyaeMaeeOBa4MaeeizaqMaeeiiaaIae8NSdiMaeyypa0Zaa8qmaeaacqWGMbGzdaWgaaWcbaGaemizaqgabeaakiabcIcaOiabdIha4jabcMcaPiabdsgaKjabdIha4jabg2da9iabigdaXiabgkHiTiabdsfaujabdchaWjabc6caUiaaxMaacaWLjaWaaeWaaeaacqaIYaGmaiaawIcacaGLPaaaaSqaaiabgkHiTiabg6HiLcqaaiabdQha6bqdcqGHRiI8aaWcbaGaemOEaOhabaGaeyOhIukaniabgUIiYdaaaa@61EC@

Then the probability of a consistent pair becomes

*p*_*con *_= (1 - *α*)(1 - *β*),

and the probability of a tied pair becomes

*p*_*tied *_= (1 - *α*) *β *+ *α *(1 - *β*).

Assigning these probabilities into (1), we have

*C *= 1 - (*α *+ *β*)/2.     (3)

It follows that the highest pair consistency probability is achieved when the sum of the two types of errors, *α *+ *β*, is minimized. Since *sensitivity *is (1 - *β*) and *specificity *is (1 - *α*), we have

*C *= (*sensitivity *+ *specificity*)/2.     (4)

Therefore the highest pair consistency probability is achieved when the sum of sensitivity and specificity is maximized.

**Figure 1:**
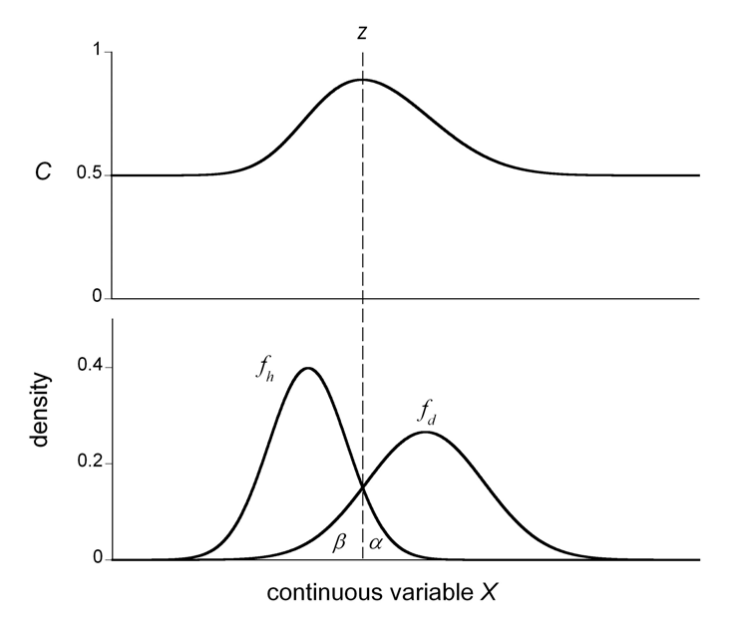
**Sample illustration of the change of pair consistency probability *C***. *Lower curves*: sample illustration of the probability density functions in the healthy group (*f*_*h*_) and in the diseased group (*f*_*d*_); *Upper curve*: pair consistency probability *C *(=(1- (*α *+ *β*)/2)) as a function of cutoff point *z*. The sum of the two types of errors, *α *+ *β*, takes a local minimum at the point where *f*_*h *_and *f*_*d *_intersect.

Figure 1 illustrates the changes of *C *when *f*_*h *_and *f*_*d *_are normal. Let *z *be the cutoff point where *f*_*h *_and *f*_*d *_cross between two peaks. If the cutoff point is shifted to the right from *z*, then *α *will decrease and *β *will increase. In this case, since *f*_*d *_is greater than *f*_*h *_in this interval, the increase of *β *is greater than the decrease of *α*. If the cutoff point is shifted to the left, then the opposite is true. Therefore, the sum of the two types of errors, *α *+ *β*, occupies the local minimum at the point where *f*_*h *_and *f*_*d *_intersect between the peaks. If *f*_*h *_and *f*_*d *_are unimodal and cross only at one point, *α *+ *β *occupies the true minimum at the cross point.

### Generation and meaning of the ROC straight line graph for a dichotomous variable

**Figure 2:**
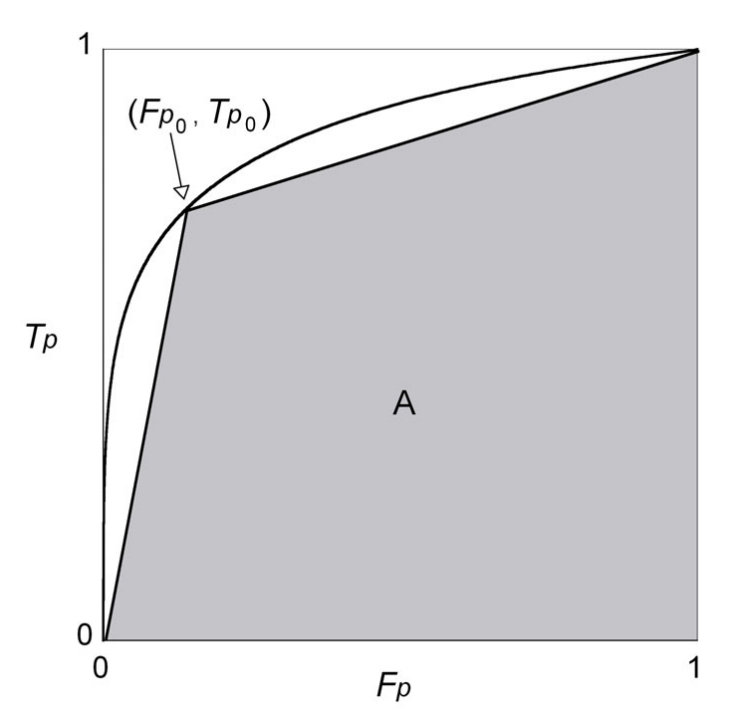
**The ROC curve and ROC straight line graph for the sample distributions in Figure 1**. The ROC curve was derived from the distributions in Figure 1 and a ROC straight line graph for the cutoff point *z*_0_, which gives the maximum *C*, was also plotted. Filled part *A *shows the area under the ROC straight line graph.

As we have described earlier, when the independent variable is continuous, *Tp *and *Fp *both decrease continuously and the ROC curve can be depicted as the trace of points (*Fp *, *Tp *). But what happens to the ROC curve when the variable is dichotomous? Let *z*_0 _represent the cutoff point and *Fp*_0 _and *Tp*_0 _denote the false positive and true positive fractions for *z*_0_, respectively. Unlike the continuous variables, only three points (1, 1), (*Fp*_0_, *Tp*_0_) and (0, 0) are depicted in *Fp *- *Tp *coordinates and we cannot obtain a true curve (see Figure 2). We jointed these points with straight lines, and labelled this graph the *ROC straight line graph*. Then area *A *under the ROC straight line graph becomes:

*A *= *Fp*_0_*Tp*_0_/2 + (1 - *Fp*_0_)*Tp*_0 _+ (1 - *Fp*_0_)(1 - *Tp*_0_)/2

= 1 - (*α *+ *β*)/2 = *C*.     (5)

This means that for a dichotomous variable, the area under the *ROC straight line graph *for a dichotomous variable is, analogous to the case with a continuous variable, equivalent to the pair consistency probability *C*. Therefore, finding a cutoff point that maximizes *C *is equivalent to the problem of finding the point (*Fp*_0 _, *Tp*_0 _) on the original ROC *curve *that maximizes the area *A *under the ROC *straight line *graph.

### Optimal cutoff points for polychotomization

Next, consider the polychotomous case. Again, let *x*_[*h*] _be a sample from the continuous random variable *X *in the healthy group and *x*_[*d*] _a sample from the same variable in the disease group, both taken randomly. Let *z*_0 _= -∞, *z*_*n *_= ∞ and *z*_1_, *z*_2_,..., *z*_*n*-1 _be cutoff points where *z*_1_<*z*_2 _<...<*z*_*n*-1_. We define that

Hk=P(zk−1<x[h]<zk)=∫zk−1zkfh(x)dx(k=1,...,n)
 MathType@MTEF@5@5@+=feaafiart1ev1aaatCvAUfKttLearuWrP9MDH5MBPbIqV92AaeXatLxBI9gBaebbnrfifHhDYfgasaacH8akY=wiFfYdH8Gipec8Eeeu0xXdbba9frFj0=OqFfea0dXdd9vqai=hGuQ8kuc9pgc9s8qqaq=dirpe0xb9q8qiLsFr0=vr0=vr0dc8meaabaqaciaacaGaaeqabaqabeGadaaakeaafaqabeqacaaabaGaemisaG0aaSbaaSqaaiabdUgaRbqabaGccqGH9aqpcqWGqbaucqGGOaakcqWG6bGEdaWgaaWcbaGaem4AaSMaeyOeI0IaeGymaedabeaakiabgYda8iabdIha4naaBaaaleaacqGGBbWwcqWGObaAcqGGDbqxaeqaaOGaeyipaWJaemOEaO3aaSbaaSqaaiabdUgaRbqabaGccqGGPaqkcqGH9aqpdaWdXaqaaiabdAgaMnaaBaaaleaacqWGObaAaeqaaOGaeiikaGIaemiEaGNaeiykaKcaleaacqWG6bGEdaWgaaadbaGaem4AaSMaeyOeI0IaeGymaedabeaaaSqaaiabdQha6naaBaaameaacqWGRbWAaeqaaaqdcqGHRiI8aOGaemizaqMaemiEaGhabaGaeiikaGIaem4AaSMaeyypa0JaeGymaeJaeiilaWIaeiOla4IaeiOla4IaeiOla4IaeiilaWIaemOBa4MaeiykaKcaaaaa@61BD@

Dk=P(zk−1<x[d]<zk)=∫zk−1zkfd(x)dx(k=1,...,n).
 MathType@MTEF@5@5@+=feaafiart1ev1aaatCvAUfKttLearuWrP9MDH5MBPbIqV92AaeXatLxBI9gBaebbnrfifHhDYfgasaacH8akY=wiFfYdH8Gipec8Eeeu0xXdbba9frFj0=OqFfea0dXdd9vqai=hGuQ8kuc9pgc9s8qqaq=dirpe0xb9q8qiLsFr0=vr0=vr0dc8meaabaqaciaacaGaaeqabaqabeGadaaakeaafaqabeqacaaabaGaemiraq0aaSbaaSqaaiabdUgaRbqabaGccqGH9aqpcqWGqbaucqGGOaakcqWG6bGEdaWgaaWcbaGaem4AaSMaeyOeI0IaeGymaedabeaakiabgYda8iabdIha4naaBaaaleaacqGGBbWwcqWGKbazcqGGDbqxaeqaaOGaeyipaWJaemOEaO3aaSbaaSqaaiabdUgaRbqabaGccqGGPaqkcqGH9aqpdaWdXaqaaiabdAgaMnaaBaaaleaacqWGKbazaeqaaOGaeiikaGIaemiEaGNaeiykaKcaleaacqWG6bGEdaWgaaadbaGaem4AaSMaeyOeI0IaeGymaedabeaaaSqaaiabdQha6naaBaaameaacqWGRbWAaeqaaaqdcqGHRiI8aOGaemizaqMaemiEaGhabaGaeiikaGIaem4AaSMaeyypa0JaeGymaeJaeiilaWIaeiOla4IaeiOla4IaeiOla4IaeiilaWIaemOBa4MaeiykaKIaeiOla4caaaaa@6289@

Then the probabilities for tied and concordant pairs become

ptied=∑k=1nHk⋅Dk and pcon=∑k=1n−1Hk⋅(∑j=k+1nDj),
 MathType@MTEF@5@5@+=feaafiart1ev1aaatCvAUfKttLearuWrP9MDH5MBPbIqV92AaeXatLxBI9gBaebbnrfifHhDYfgasaacH8akY=wiFfYdH8Gipec8Eeeu0xXdbba9frFj0=OqFfea0dXdd9vqai=hGuQ8kuc9pgc9s8qqaq=dirpe0xb9q8qiLsFr0=vr0=vr0dc8meaabaqaciaacaGaaeqabaqabeGadaaakeaacqWGWbaCdaWgaaWcbaGaemiDaqNaemyAaKMaemyzauMaemizaqgabeaakiabg2da9maaqahabaGaemisaG0aaSbaaSqaaiabdUgaRbqabaGccqGHflY1cqWGebardaWgaaWcbaGaem4AaSgabeaaaeaacqWGRbWAcqGH9aqpcqaIXaqmaeaacqWGUbGBa0GaeyyeIuoakiabbccaGiabbggaHjabb6gaUjabbsgaKjabbccaGiabdchaWnaaBaaaleaacqWGJbWycqWGVbWBcqWGUbGBaeqaaOGaeyypa0ZaaabCaeaacqWGibasdaWgaaWcbaGaem4AaSgabeaakiabgwSixpaabmaabaWaaabCaeaacqWGebardaWgaaWcbaGaemOAaOgabeaaaeaacqWGQbGAcqGH9aqpcqWGRbWAcqGHRaWkcqaIXaqmaeaacqWGUbGBa0GaeyyeIuoaaOGaayjkaiaawMcaaaWcbaGaem4AaSMaeyypa0JaeGymaedabaGaemOBa4MaeyOeI0IaeGymaedaniabggHiLdGccqGGSaalaaa@6BB1@

and the pair consistency probability *C *can be calculated from equation (1).

We also define

*Tp*_*k *_= *P *(*x*_[*d*] _> *z*_*k*_) and *Fp*_*k *_= *P *(*x*_[*h*] _> *z*_*k*_)     (*k *= 0,..., *n*).

**Figure 3:**
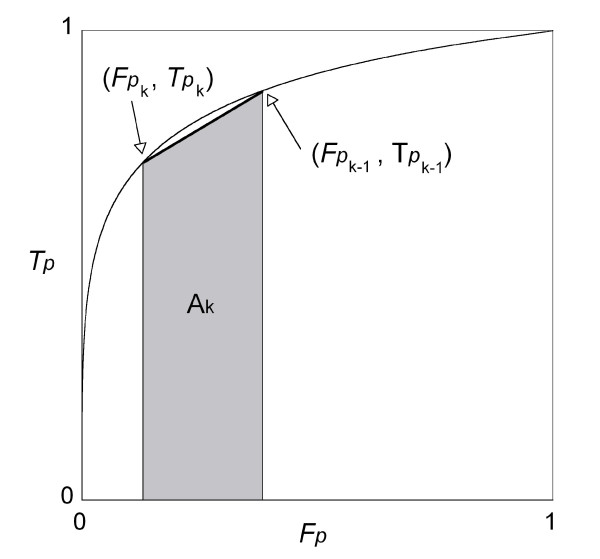
**Area *A*_*k *_under the ROC straight line graph**. The filled part shows the area *A*_*k *_under the ROC straight line graph with end points (*Fp*_*k*-1_, *Tp*_*k*-1_) and (*Fp*_*k*_, *Tp*_*k*_).

The points (*Fp*_*k*_, *Tp*_*k*_) lie on the original ROC curve, and the set of points (*Fp*_*k*_, *Tp*_*k*_) jointed by straight lines yields the ROC straight line graph. Let *A *represent the area under the ROC straight line graph and *A*_*k *_represent the area under the line whose ends are (*Fp*_*k*-1_, *Tp*_*k*-1_) and (*Fp*_*k*_, *Tp*_*k*_). As illustrated in Figure 3, the area *A*_*k *_is

Ak=12{Fpk−1−Fpk}⋅{Tpk−1+Tpk}.
 MathType@MTEF@5@5@+=feaafiart1ev1aaatCvAUfKttLearuWrP9MDH5MBPbIqV92AaeXatLxBI9gBaebbnrfifHhDYfgasaacH8akY=wiFfYdH8Gipec8Eeeu0xXdbba9frFj0=OqFfea0dXdd9vqai=hGuQ8kuc9pgc9s8qqaq=dirpe0xb9q8qiLsFr0=vr0=vr0dc8meaabaqaciaacaGaaeqabaqabeGadaaakeaacqWGbbqqdaWgaaWcbaGaem4AaSgabeaakiabg2da9maalaaabaGaeGymaedabaGaeGOmaidaaiabcUha7jabdAeagjabdchaWnaaBaaaleaacqWGRbWAcqGHsislcqaIXaqmaeqaaOGaeyOeI0IaemOrayKaemiCaa3aaSbaaSqaaiabdUgaRbqabaGccqGG9bqFcqGHflY1cqGG7bWEcqWGubavcqWGWbaCdaWgaaWcbaGaem4AaSMaeyOeI0IaeGymaedabeaakiabgUcaRiabdsfaujabdchaWnaaBaaaleaacqWGRbWAaeqaaOGaeiyFa0NaeiOla4caaa@517F@

Therefore,

Ak=12P(zk−1<x[h]<zk)⋅{P(x[d]>zk−1)+P(x[d]>zk)}=P(zk−1<x[h]<zk)⋅P(x[d]>zk)+12P(zk−1<x[h]<zk)⋅P(zk−1<x[d]<zk)={Hk⋅(∑j=k+1nDj)+12Hk⋅Dk(k=1,...,n−1)12Hn⋅Dn(k=n)
 MathType@MTEF@5@5@+=feaafiart1ev1aaatCvAUfKttLearuWrP9MDH5MBPbIqV92AaeXatLxBI9gBaebbnrfifHhDYfgasaacH8akY=wiFfYdH8Gipec8Eeeu0xXdbba9frFj0=OqFfea0dXdd9vqai=hGuQ8kuc9pgc9s8qqaq=dirpe0xb9q8qiLsFr0=vr0=vr0dc8meaabaqaciaacaGaaeqabaqabeGadaaakqaabeqaaiabdgeabnaaBaaaleaacqWGRbWAaeqaaOGaeyypa0ZaaSaaaeaacqaIXaqmaeaacqaIYaGmaaGaemiuaaLaeiikaGIaemOEaO3aaSbaaSqaaiabdUgaRjabgkHiTiabigdaXaqabaGccqGH8aapcqWG4baEdaWgaaWcbaGaei4waSLaemiAaGMaeiyxa0fabeaakiabgYda8iabdQha6naaBaaaleaacqWGRbWAaeqaaOGaeiykaKIaeyyXICTaei4EaSNaemiuaaLaeiikaGIaemiEaG3aaSbaaSqaaiabcUfaBjabdsgaKjabc2faDbqabaGccqGH+aGpcqWG6bGEdaWgaaWcbaGaem4AaSMaeyOeI0IaeGymaedabeaakiabcMcaPiabgUcaRiabdcfaqjabcIcaOiabdIha4naaBaaaleaacqGGBbWwcqWGKbazcqGGDbqxaeqaaOGaeyOpa4JaemOEaO3aaSbaaSqaaiabdUgaRbqabaGccqGGPaqkcqGG9bqFaeaacqGH9aqpcqWGqbaucqGGOaakcqWG6bGEdaWgaaWcbaGaem4AaSMaeyOeI0IaeGymaedabeaakiabgYda8iabdIha4naaBaaaleaacqGGBbWwcqWGObaAcqGGDbqxaeqaaOGaeyipaWJaemOEaO3aaSbaaSqaaiabdUgaRbqabaGccqGGPaqkcqGHflY1cqWGqbaucqGGOaakcqWG4baEdaWgaaWcbaGaei4waSLaemizaqMaeiyxa0fabeaakiabg6da+iabdQha6naaBaaaleaacqWGRbWAaeqaaOGaeiykaKIaey4kaSYaaSaaaeaacqaIXaqmaeaacqaIYaGmaaGaemiuaaLaeiikaGIaemOEaO3aaSbaaSqaaiabdUgaRjabgkHiTiabigdaXaqabaGccqGH8aapcqWG4baEdaWgaaWcbaGaei4waSLaemiAaGMaeiyxa0fabeaakiabgYda8iabdQha6naaBaaaleaacqWGRbWAaeqaaOGaeiykaKIaeyyXICTaemiuaaLaeiikaGIaemOEaO3aaSbaaSqaaiabdUgaRjabgkHiTiabigdaXaqabaGccqGH8aapcqWG4baEdaWgaaWcbaGaei4waSLaemizaqMaeiyxa0fabeaakiabgYda8iabdQha6naaBaaaleaacqWGRbWAaeqaaOGaeiykaKcabaGaeyypa0ZaaiqabeaafaqaaeGacaaabaGaemisaG0aaSbaaSqaaiabdUgaRbqabaGccqGHflY1daqadaqaamaaqahabaGaemiraq0aaSbaaSqaaiabdQgaQbqabaaabaGaemOAaOMaeyypa0Jaem4AaSMaey4kaSIaeGymaedabaGaemOBa4ganiabggHiLdaakiaawIcacaGLPaaacqGHRaWkdaWcaaqaaiabigdaXaqaaiabikdaYaaacqWGibasdaWgaaWcbaGaem4AaSgabeaakiabgwSixlabdseaenaaBaaaleaacqWGRbWAaeqaaaGcbaGaeiikaGIaem4AaSMaeyypa0JaeGymaeJaeiilaWIaeiOla4IaeiOla4IaeiOla4IaeiilaWIaemOBa4MaeyOeI0IaeGymaeJaeiykaKcabaWaaSaaaeaacqaIXaqmaeaacqaIYaGmaaGaemisaG0aaSbaaSqaaiabd6gaUbqabaGccqGHflY1cqWGebardaWgaaWcbaGaemOBa4gabeaaaOqaaiabcIcaOiabdUgaRjabg2da9iabd6gaUjabcMcaPaaaaiaawUhaaaaaaa@EC8E@

Then we have

A=∑k=1nAk=∑k=1n−1Hk⋅(∑j=k+1nDj)+12∑k=1nHk⋅Dk=pcon+12ptied=C.     (6)
 MathType@MTEF@5@5@+=feaafiart1ev1aaatCvAUfKttLearuWrP9MDH5MBPbIqV92AaeXatLxBI9gBaebbnrfifHhDYfgasaacH8akY=wiFfYdH8Gipec8Eeeu0xXdbba9frFj0=OqFfea0dXdd9vqai=hGuQ8kuc9pgc9s8qqaq=dirpe0xb9q8qiLsFr0=vr0=vr0dc8meaabaqaciaacaGaaeqabaqabeGadaaakeaafaqadeWabaaabaGaemyqaeKaeyypa0ZaaabCaeaacqWGbbqqdaWgaaWcbaGaem4AaSgabeaaaeaacqWGRbWAcqGH9aqpcqaIXaqmaeaacqWGUbGBa0GaeyyeIuoaaOqaaiabg2da9maaqahabaGaemisaG0aaSbaaSqaaiabdUgaRbqabaGccqGHflY1daqadaqaamaaqahabaGaemiraq0aaSbaaSqaaiabdQgaQbqabaaabaGaemOAaOMaeyypa0Jaem4AaSMaey4kaSIaeGymaedabaGaemOBa4ganiabggHiLdaakiaawIcacaGLPaaacqGHRaWkdaWcaaqaaiabigdaXaqaaiabikdaYaaadaaeWbqaaiabdIeainaaBaaaleaacqWGRbWAaeqaaOGaeyyXICTaemiraq0aaSbaaSqaaiabdUgaRbqabaaabaGaem4AaSMaeyypa0JaeGymaedabaGaemOBa4ganiabggHiLdaaleaacqWGRbWAcqGH9aqpcqaIXaqmaeaacqWGUbGBcqGHsislcqaIXaqma0GaeyyeIuoaaOqaaiabg2da9iabdchaWnaaBaaaleaacqWGJbWycqWGVbWBcqWGUbGBaeqaaOGaey4kaSYaaSaaaeaacqaIXaqmaeaacqaIYaGmaaGaemiCaa3aaSbaaSqaaiabdsha0jabdMgaPjabdwgaLjabdsgaKbqabaGccqGH9aqpcqWGdbWqcqGGUaGlaaGaaCzcaiaaxMaadaqadaqaaiabiAda2aGaayjkaiaawMcaaaaa@7D59@

Again, the pair consistency probability *C *for the polychotomized variable is equivalent to the area under its ROC straight line graph, and the problem of finding the optimal cutoff points that maximize *C *is mathematically equivalent to finding the set of edge points of the ROC straight line graph that maximizes the area *A *under that graph.

### Optimal cutoff points for variables for which normal and diseased cases have a common central tendency

There are many predictive variables whose central values refer to a normal state and whose more dispersed values refer to less preferable states. In the example of rhabdomyolysis prognosis that will follow later, body temperature, pulse rate, plasma sodium, and plasma pH are such variables. For these predictors, we need to find at least two cutoff points to discriminate normal and abnormal states. If we denote the values of the cutoff points *z*_1 _and *z*_2 _(*z*_1 _<*z*_2_), and regard the value between these two cutoff points as normal, then type I error *α *and type II error *β *become:

α=∫−∞z1fh(x)dx+∫z2∞fh(x)dx=Fp
 MathType@MTEF@5@5@+=feaafiart1ev1aaatCvAUfKttLearuWrP9MDH5MBPbIqV92AaeXatLxBI9gBaebbnrfifHhDYfgasaacH8akY=wiFfYdH8Gipec8Eeeu0xXdbba9frFj0=OqFfea0dXdd9vqai=hGuQ8kuc9pgc9s8qqaq=dirpe0xb9q8qiLsFr0=vr0=vr0dc8meaabaqaciaacaGaaeqabaqabeGadaaakeaaiiGacqWFXoqycqGH9aqpdaWdXaqaaiabdAgaMnaaBaaaleaacqWGObaAaeqaaOGaeiikaGIaemiEaGNaeiykaKcaleaacqGHsislcqGHEisPaeaacqWG6bGEdaWgaaadbaGaeGymaedabeaaa0Gaey4kIipakiabdsgaKjabdIha4jabgUcaRmaapedabaGaemOzay2aaSbaaSqaaiabdIgaObqabaGccqGGOaakcqWG4baEcqGGPaqkaSqaaiabdQha6naaBaaameaacqaIYaGmaeqaaaWcbaGaeyOhIukaniabgUIiYdGccqWGKbazcqWG4baEcqGH9aqpcqWGgbGrcqWGWbaCaaa@52E6@

and

β=∫z1z2fd(x)dx=1−Tp.
 MathType@MTEF@5@5@+=feaafiart1ev1aaatCvAUfKttLearuWrP9MDH5MBPbIqV92AaeXatLxBI9gBaebbnrfifHhDYfgasaacH8akY=wiFfYdH8Gipec8Eeeu0xXdbba9frFj0=OqFfea0dXdd9vqai=hGuQ8kuc9pgc9s8qqaq=dirpe0xb9q8qiLsFr0=vr0=vr0dc8meaabaqaciaacaGaaeqabaqabeGadaaakeaaiiGacqWFYoGycqGH9aqpdaWdXaqaaiabdAgaMnaaBaaaleaacqWGKbazaeqaaOGaeiikaGIaemiEaGNaeiykaKcaleaacqWG6bGEdaWgaaadbaGaeGymaedabeaaaSqaaiabdQha6naaBaaameaacqaIYaGmaeqaaaqdcqGHRiI8aOGaemizaqMaemiEaGNaeyypa0JaeGymaeJaeyOeI0IaemivaqLaemiCaaNaeiOla4caaa@4600@

The pair consistency probability *C *can now be calculated with equation (3) and the combination of cutoff points (*z*_1_, *z*_2_) which maximizes (3) becomes the solution. In case of categorization of the variable into more than three states, we can define the optimal combination of cutoff points as follows: Let *z*_*n *_= -∞, *w*_*n *_= ∞ and *z*_1_, *z*_2_,..., *z*_*n*-1_, *w*_1_, *w*_2_,..., *w*_*n*-1 _be cutoff points where z_*n*-1 _<...<*z*_2 _<*z*_1 _<*w*_1 _<*w*_2 _<...<*w*_*n*-1_, and

H1=∫z1w1fh(x)dx,D1=∫z1w1fd(x)dx
 MathType@MTEF@5@5@+=feaafiart1ev1aaatCvAUfKttLearuWrP9MDH5MBPbIqV92AaeXatLxBI9gBaebbnrfifHhDYfgasaacH8akY=wiFfYdH8Gipec8Eeeu0xXdbba9frFj0=OqFfea0dXdd9vqai=hGuQ8kuc9pgc9s8qqaq=dirpe0xb9q8qiLsFr0=vr0=vr0dc8meaabaqaciaacaGaaeqabaqabeGadaaakeaafaqabeqacaaabaGaemisaG0aaSbaaSqaaiabigdaXaqabaGccqGH9aqpdaWdXaqaaiabdAgaMnaaBaaaleaacqWGObaAaeqaaOGaeiikaGIaemiEaGNaeiykaKIaemizaqMaemiEaGNaeiilaWcaleaacqWG6bGEdaWgaaadbaGaeGymaedabeaaaSqaaiabdEha3naaBaaameaacqaIXaqmaeqaaaqdcqGHRiI8aaGcbaGaemiraq0aaSbaaSqaaiabigdaXaqabaGccqGH9aqpdaWdXaqaaiabdAgaMnaaBaaaleaacqWGKbazaeqaaOGaeiikaGIaemiEaGNaeiykaKIaemizaqMaemiEaGhaleaacqWG6bGEdaWgaaadbaGaeGymaedabeaaaSqaaiabdEha3naaBaaameaacqaIXaqmaeqaaaqdcqGHRiI8aaaaaaa@5493@

Hk=∫zkzk−1fh(x)dx+∫wk−1wkfh(x)dx(k=2,...,n),
 MathType@MTEF@5@5@+=feaafiart1ev1aaatCvAUfKttLearuWrP9MDH5MBPbIqV92AaeXatLxBI9gBaebbnrfifHhDYfgasaacH8akY=wiFfYdH8Gipec8Eeeu0xXdbba9frFj0=OqFfea0dXdd9vqai=hGuQ8kuc9pgc9s8qqaq=dirpe0xb9q8qiLsFr0=vr0=vr0dc8meaabaqaciaacaGaaeqabaqabeGadaaakeaafaqabeqacaaabaGaemisaG0aaSbaaSqaaiabdUgaRbqabaGccqGH9aqpdaWdXaqaaiabdAgaMnaaBaaaleaacqWGObaAaeqaaOGaeiikaGIaemiEaGNaeiykaKIaemizaqMaemiEaGNaey4kaSYaa8qmaeaacqWGMbGzdaWgaaWcbaGaemiAaGgabeaakiabcIcaOiabdIha4jabcMcaPiabdsgaKjabdIha4bWcbaGaem4DaC3aaSbaaWqaaiabdUgaRjabgkHiTiabigdaXaqabaaaleaacqWG3bWDdaWgaaadbaGaem4AaSgabeaaa0Gaey4kIipaaSqaaiabdQha6naaBaaameaacqWGRbWAaeqaaaWcbaGaemOEaO3aaSbaaWqaaiabdUgaRjabgkHiTiabigdaXaqabaaaniabgUIiYdaakeaacqGGOaakcqWGRbWAcqGH9aqpcqaIYaGmcqGGSaalcqGGUaGlcqGGUaGlcqGGUaGlcqGGSaalcqWGUbGBcqGGPaqkcqGGSaalaaaaaa@62FF@

Dk=∫zkzk−1fd(x)dx+∫wk−1wkfd(x)dx(k=2,...,n).
 MathType@MTEF@5@5@+=feaafiart1ev1aaatCvAUfKttLearuWrP9MDH5MBPbIqV92AaeXatLxBI9gBaebbnrfifHhDYfgasaacH8akY=wiFfYdH8Gipec8Eeeu0xXdbba9frFj0=OqFfea0dXdd9vqai=hGuQ8kuc9pgc9s8qqaq=dirpe0xb9q8qiLsFr0=vr0=vr0dc8meaabaqaciaacaGaaeqabaqabeGadaaakeaafaqabeqacaaabaGaemiraq0aaSbaaSqaaiabdUgaRbqabaGccqGH9aqpdaWdXaqaaiabdAgaMnaaBaaaleaacqWGKbazaeqaaOGaeiikaGIaemiEaGNaeiykaKIaemizaqMaemiEaGNaey4kaSYaa8qmaeaacqWGMbGzdaWgaaWcbaGaemizaqgabeaakiabcIcaOiabdIha4jabcMcaPiabdsgaKjabdIha4bWcbaGaem4DaC3aaSbaaWqaaiabdUgaRjabgkHiTiabigdaXaqabaaaleaacqWG3bWDdaWgaaadbaGaem4AaSgabeaaa0Gaey4kIipaaSqaaiabdQha6naaBaaameaacqWGRbWAaeqaaaWcbaGaemOEaO3aaSbaaWqaaiabdUgaRjabgkHiTiabigdaXaqabaaaniabgUIiYdaakeaacqGGOaakcqWGRbWAcqGH9aqpcqaIYaGmcqGGSaalcqGGUaGlcqGGUaGlcqGGUaGlcqGGSaalcqWGUbGBcqGGPaqkcqGGUaGlaaaaaa@62EB@

Then the probabilities for tied and concordant pairs become

ptied=∑k=1nHk⋅Dk and pcon=∑k=1n−1Hk⋅(∑j=k+1nDj),
 MathType@MTEF@5@5@+=feaafiart1ev1aaatCvAUfKttLearuWrP9MDH5MBPbIqV92AaeXatLxBI9gBaebbnrfifHhDYfgasaacH8akY=wiFfYdH8Gipec8Eeeu0xXdbba9frFj0=OqFfea0dXdd9vqai=hGuQ8kuc9pgc9s8qqaq=dirpe0xb9q8qiLsFr0=vr0=vr0dc8meaabaqaciaacaGaaeqabaqabeGadaaakeaacqWGWbaCdaWgaaWcbaGaemiDaqNaemyAaKMaemyzauMaemizaqgabeaakiabg2da9maaqahabaGaemisaG0aaSbaaSqaaiabdUgaRbqabaGccqGHflY1cqWGebardaWgaaWcbaGaem4AaSgabeaaaeaacqWGRbWAcqGH9aqpcqaIXaqmaeaacqWGUbGBa0GaeyyeIuoakiabbccaGiabbggaHjabb6gaUjabbsgaKjabbccaGiabdchaWnaaBaaaleaacqWGJbWycqWGVbWBcqWGUbGBaeqaaOGaeyypa0ZaaabCaeaacqWGibasdaWgaaWcbaGaem4AaSgabeaakiabgwSixpaabmaabaWaaabCaeaacqWGebardaWgaaWcbaGaemOAaOgabeaaaeaacqWGQbGAcqGH9aqpcqWGRbWAcqGHRaWkcqaIXaqmaeaacqWGUbGBa0GaeyyeIuoaaOGaayjkaiaawMcaaaWcbaGaem4AaSMaeyypa0JaeGymaedabaGaemOBa4MaeyOeI0IaeGymaedaniabggHiLdGccqGGSaalaaa@6BB1@

and the pair consistency probability *C *can be calculated from equation (1). The combination of cutoff points that maximizes *C *becomes the solution.

### Parametric method for estimating cutoff points and predictive performance

The polychotomization methods proposed in the previous sections have been developed under conditions where the exact distribution of a prognostic or diagnostic factor in a population is known. However, in research practice we work with samples and we need to discuss whether our methods can be applied in situations involving parameter uncertainty. Although some methods were developed for correct estimation of the pair consistency probability *C *in these situations, including non-parametric ones [22-24], none of them addressed the estimation of cutoff points and they can therefore not be applied to our setting.

The challenge we are faced with is that if we repeat the evaluation of the pair consistency probability to find optimal cutoff points, for instance by increasing the possible value of the cutoff point with a certain step, it gives rise to estimation error just like the minimum *p*-value approach [9-18] and would mistakenly lead to an optimistic conclusion on the predictive performance of the model in future observations.

It is clear that we need a practical method that does not suffer from this over-estimation bias. In this paper we show that if *f*_*h *_and *f*_*d *_can be transformed to normal distributions, a parametric method provides essentially unbiased estimators of predictive performance and cutoff points.

Our method is based on the following:

a) the assumption that the probability density functions of an independent variable on the healthy and disease groups, *f*_*h *_and *f*_*d*_, are both normally distributed or can be transformed to a normal distribution,

b) the estimation of the means and standard deviations of *f*_*h *_and *f*_*d*_, *m*_*h*_, *s*_*h*_, *m*_*d*_, and *s*_*d *_from sample data,

c) the localization of the *optimal *cutoff points based on the estimated distributions f˜h
 MathType@MTEF@5@5@+=feaafiart1ev1aaatCvAUfKttLearuWrP9MDH5MBPbIqV92AaeXatLxBI9gBaebbnrfifHhDYfgasaacH8akY=wiFfYdH8Gipec8Eeeu0xXdbba9frFj0=OqFfea0dXdd9vqai=hGuQ8kuc9pgc9s8qqaq=dirpe0xb9q8qiLsFr0=vr0=vr0dc8meaabaqaciaacaGaaeqabaqabeGadaaakeaacuWGMbGzgaacamaaBaaaleaacqWGObaAaeqaaaaa@2F95@ and f˜d
 MathType@MTEF@5@5@+=feaafiart1ev1aaatCvAUfKttLearuWrP9MDH5MBPbIqV92AaeXatLxBI9gBaebbnrfifHhDYfgasaacH8akY=wiFfYdH8Gipec8Eeeu0xXdbba9frFj0=OqFfea0dXdd9vqai=hGuQ8kuc9pgc9s8qqaq=dirpe0xb9q8qiLsFr0=vr0=vr0dc8meaabaqaciaacaGaaeqabaqabeGadaaakeaacuWGMbGzgaacamaaBaaaleaacqWGKbazaeqaaaaa@2F8D@, and

d) the calculation of the predictive performance based on the estimated cutoff points.

### Distributions of the estimators for the cutoff point and the pair consistency probability

If *f*_*h *_and *f*_*d *_are both normal and *s*_*h *_= *s*_*d*_, then the two curves intersect at *x *= (*m*_*h *_+ *m*_*d*_)/2. The pair consistency probability *C *takes the maximum value at this point as mentioned earlier. In the case that *s*_*h *_is not equal to *s*_*d*_, the two curves intersect at the following two points:

x=1(sd2−sh2){(mhsd2−mdsh2)±(mhsd2−mdsh2)2−(sd2−sh2)[(mh2sd2−md2sh2)+2sh2sd2log⁡(shsd)]}     (7)
 MathType@MTEF@5@5@+=feaafiart1ev1aaatCvAUfKttLearuWrP9MDH5MBPbIqV92AaeXatLxBI9gBaebbnrfifHhDYfgasaacH8akY=wiFfYdH8Gipec8Eeeu0xXdbba9frFj0=OqFfea0dXdd9vqai=hGuQ8kuc9pgc9s8qqaq=dirpe0xb9q8qiLsFr0=vr0=vr0dc8meaabaqaciaacaGaaeqabaqabeGadaaakeaacqWG4baEcqGH9aqpdaWcaaqaaiabigdaXaqaaiabcIcaOiabdohaZnaaDaaaleaacqWGKbazaeaacqaIYaGmaaGccqGHsislcqWGZbWCdaqhaaWcbaGaemiAaGgabaGaeGOmaidaaOGaeiykaKcaaiabcUha7jabcIcaOiabd2gaTnaaBaaaleaacqWGObaAaeqaaOGaem4Cam3aa0baaSqaaiabdsgaKbqaaiabikdaYaaakiabgkHiTiabd2gaTnaaBaaaleaacqWGKbazaeqaaOGaem4Cam3aa0baaSqaaiabdIgaObqaaiabikdaYaaakiabcMcaPiabgglaXoaakaaabaGaeiikaGIaemyBa02aaSbaaSqaaiabbIgaObqabaGccqWGZbWCdaqhaaWcbaGaeeizaqgabaGaeGOmaidaaOGaeyOeI0IaemyBa02aaSbaaSqaaiabbsgaKbqabaGccqWGZbWCdaqhaaWcbaGaeeiAaGgabaGaeGOmaidaaOGaeiykaKYaaWbaaSqabeaacqaIYaGmaaGccqGHsislcqGGOaakcqWGZbWCdaqhaaWcbaGaeeizaqgabaGaeGOmaidaaOGaeyOeI0Iaem4Cam3aa0baaSqaaiabbIgaObqaaiabikdaYaaakiabcMcaPiabcUfaBjabcIcaOiabd2gaTnaaDaaaleaacqqGObaAaeaacqaIYaGmaaGccqWGZbWCdaqhaaWcbaGaeeizaqgabaGaeGOmaidaaOGaeyOeI0IaemyBa02aa0baaSqaaiabbsgaKbqaaiabikdaYaaakiabdohaZnaaDaaaleaacqqGObaAaeaacqaIYaGmaaGccqGGPaqkcqGHRaWkcqaIYaGmcqWGZbWCdaqhaaWcbaGaeeiAaGgabaGaeGOmaidaaOGaem4Cam3aa0baaSqaaiabbsgaKbqaaiabikdaYaaakiGbcYgaSjabc+gaVjabcEgaNjabcIcaOmaalaaabaGaem4Cam3aaSbaaSqaaiabbIgaObqabaaakeaacqWGZbWCdaWgaaWcbaGaeeizaqgabeaaaaGccqGGPaqkcqGGDbqxcqGG9bqFaSqabaGccaWLjaGaaCzcamaabmaabaGaeG4naCdacaGLOaGaayzkaaaaaa@9ADA@

and the point that is located between *m*_*h *_and *m*_*d *_can be used to calculate the true maximum value of the pair consistency probability *C *with equations (2) and (3). As it is difficult to evaluate the statistical properties of the above formulae analytically, even for the simplest dichotomization case, we performed a Monte Carlo simulation to assess the estimation of the cutoff points and the corresponding *C*. For these purposes, a custom simulation program was written in the programming language Pascal with the following characteristics:

a) the assumption that *f*_*h *_and *f*_*d *_are both normal,

b) generation of samples of healthy and disease groups, each with a given number of measurements, by randomly generating the value of the prognostic variable,

c) estimation of the optimal cutoff points and pair consistency probability *C *by naïve stepwise repeated search, in which the cutoff point is changed with a certain small step *Δz *and the corresponding *C *is evaluated based on the sample data to find a point which gives the maximum *C*. In case of polychotomization, this step is iterated for every combination of possible cutoff values,

d) estimation of the parameters of *f*_*h *_and *f*_*d *_and calculation of the optimal cutoff points based on the estimated distributions (including the corresponding predictive ability *C*), in which cutoff points are searched numerically in the same manner as the above stepwise repeated search based not on the sample data but on the estimated PDFs,

e) repeat the above sample generation and estimating steps 10,000 or 100,000 times for each of various combinations of population parameters.

### Extension for multiple associated independent variables

Thus far, we have discussed a method for selecting cutoff points that maximizes the predictive ability of each prognostic variable individually. When a regression model has more than one explanatory variable, the version of our method presented in this article can only be applied if the variables are not associated (no correlation and no interaction). Since associations between prognostic variables are common, our method requires a multivariable extension in which cutoff points are found while taking such associations into account.

Our maximum *C *index approach can be applied to multivariate scenario if the distributions of a number of prognostic variables for healthy and diseased groups can be described by multivariate normal distributions and if the calculation times are acceptable [26]. However, because we are still in the process of assessing the performance of multivariable extensions and comparisons with other approaches, we will only give a short summary below:

a) determine the regression model that best fits the observations,

b) estimate the multivariate normal distribution parameters from the observed data,

c) for a set of categorized variables defined by a combination of cutoff points, calculate the regression equation and evaluate its overall *C *index (based not on the observed data but on the estimated distributions),

d) iterate (c) systematically for every combination of cutoff points and select the combination of cutoff points which gives the maximum overall *C *index for the regression equation.

## Results

### Evaluation of the parametric method by Monte Carlo simulation

In this section, we present an evaluation of our parametric method, together with the naïve application of a stepwise repeated search based on multiple evaluations. In the absence of a standard method for polychotomization, the latter is currently probably the first choice for researchers, mainly due to its simplicity.

**Figure 4:**
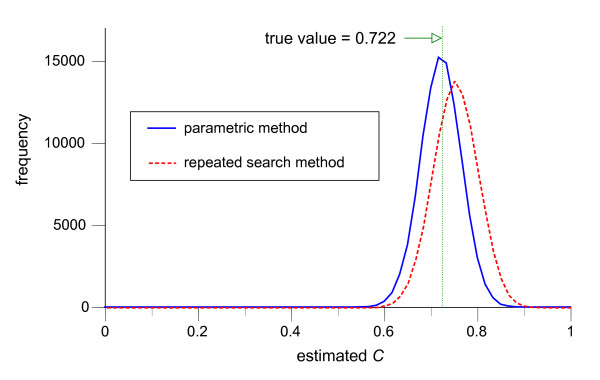
**Distributions of estimated pair consistency probability *C *in 100,000 simulations of dichotomization**. The frequency distributions of the estimate of the pair consistency probability *C *by the repeated search method (dotted line) and the parametric method (solid line) in 100,000 simulations of dichotomization, with *f*_*h *_~ *N*(0, 1^2^), *f*_*d *_~ *N*(1.5, 2^2^) and *n*_*h *_= *n*_*d *_= 30. The class width for the graph is 0.0167.

**Figure 5:**
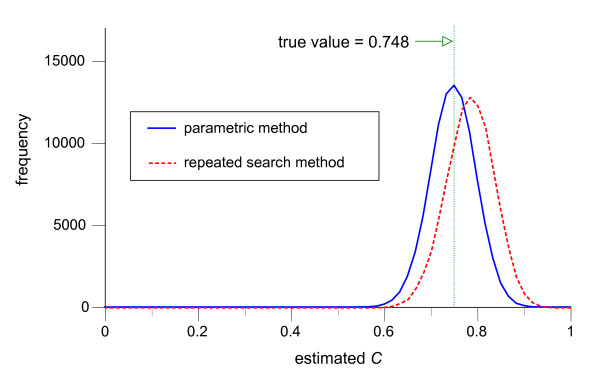
**Distributions of estimated pair consistency probability *C *in 100,000 simulations of trichotomization**. The frequency distributions of the estimate of the pair consistency probability *C *by the repeated search method (dotted line) and the parametric method (solid line) in 100,000 simulations of trichotomization, with *f*_*h *_~ *N*(0, 1^2^), *f*_*d *_~ *N*(1.5, 2^2^) and *n*_*h *_= *n*_*d *_= 30. The class width for the graph is 0.0167.

**Figure 6:**
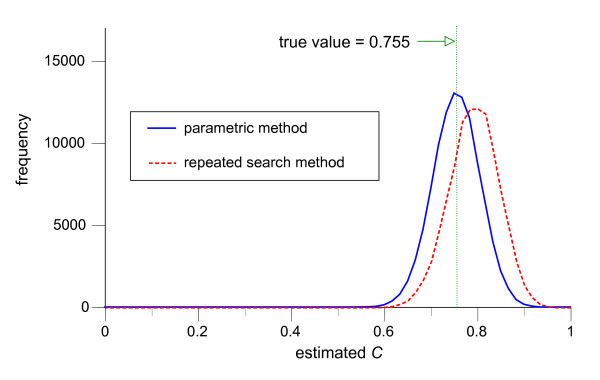
**Distributions of estimated pair consistency probability *C *in 100,000 simulations of polychotomization to four categories**. The frequency distributions of the estimate of the pair consistency probability *C *by the repeated search method (dotted line) and the parametric method (solid line) in 100,000 simulations of polychotomization to four categories, with *f*_*h *_~ *N*(0, 1^2^), *f*_*d *_~ *N*(1.5, 2^2^) and *n*_*h *_= *n*_*d *_= 30. The class width for the graph is 0.0167.

Figures 4, 5, 6, illustrate the frequency distributions of the estimates of predictive performance *C *for the repeated search method and the parametric method for dichotomization (Figure 4), trichotomization (Figure 5), and polychotomization into four categories (Figure 6), when *f*_*h *_and *f*_*d *_are both normally distributed and *n*_*h *_= *n*_*d *_= 30. Since the true values for the *C *were 0.722, 0.748 and 0.755 for dichotomization, trichotomization and polychotomization into four categories, the parametric method provides essentially unbiased normally distributed estimators (means and SDs: 0.725 ± 0.043, 0.751 ± 0.048, and 0.758 ± 0.050), whereas the repeated search method has relatively large positive biases (0.752 ± 0.048, 0.786 ± 0.051, and 0.795 ± 0.053).

**Figure 7:**
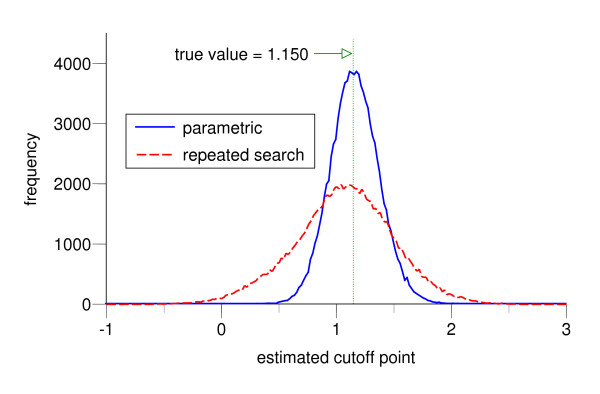
**The frequency distributions of the estimated optimal cutoff point**. The frequency distributions of the optimal cutoff points estimated by the repeated search method (dotted line) and the parametric method (solid line) in 100,000 simulations of dichotomization for the same case in Figure 4 with *f*_*h *_~ *N*(0, 1^2^), *f*_*d *_~ *N*(1.5, 2^2^) and *n*_*h *_= *n*_*d *_= 30. The class width for the graph is 0.02.

Figure 7 shows the frequencies of the optimal cutoff point in dichotomization estimated by the each of two methods. Whereas the true cutoff point is 1.150, the estimated values and their standard deviations are 1.175 ± 0.209 with the parametric approach and 1.071 ± 0.433 with the repeated search method, which means the former provides a more accurate estimator for the cutoff point with higher precision.

**Figure 8:**
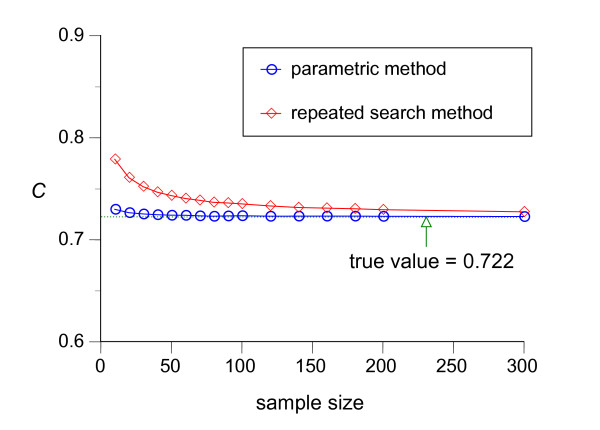
**Changes of estimated pair consistency probability *C *in dichotomization as a function of sample size**. Results from Monte Carlo simulation of the changes of the mean value of the estimated pair consistency probability *C *by the repeated search method (red line with squares) and the parametric method (blue line with circles) for various sample sizes each of which is calculated by 10,000 simulations of dichotomization with *f*_*h *_~ *N*(0, 1^2^) and *f*_*d *_~ *N*(1.5, 2^2^).

**Figure 9:**
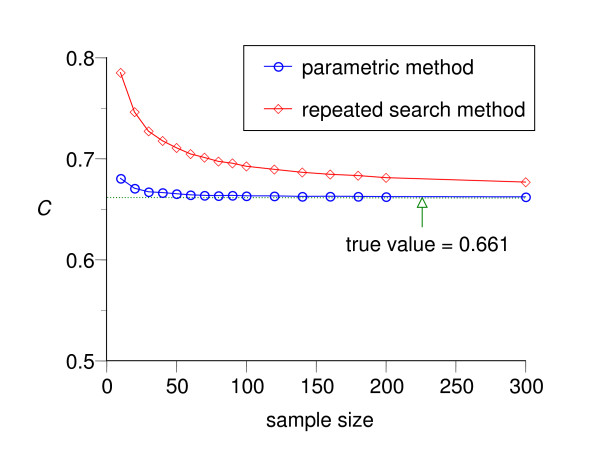
**Changes of estimated pair consistency probability *C *in trichotomization as a function of sample size**. The changes of the mean value of the estimated *C *for various sample sizes each of which is calculated by 10,000 simulations of trichotomization for a variable with a common central tendency with *f*_*h *_~ *N*(0, 1^2^) and *f*_*d *_~ *N*(0, 2^2^).

We repeated the above simulations for various *n*_*h *_and *n*_*d *_(*n*_*h *_= *n*_*d*_) and Figure 8 and Figure 9 summarize the results. The graphs show that the estimation by the parametric method is almost unbiased even if the sample size is relatively small, both for dichotomization (Figure 8) and trichotomization for variables whose realizations in healthy and diseased groups have a similar central tendency (Figure 9), whereas the naïve repeated search method shows non-negligible bias even when the sample size is large (*n *= 300).

### Distributions of estimators from the parametric method

Table 1 shows how the pair consistency probability *C *increases when the number of the cutoff point changes from one to three for the case that *x*_[*h*]_~ *N*(0, 1^2^) and *x*_[*d*] _~ *N*(*μ*_*d*_, 1.5^2^). For instance, when the pair consistency probability for the original continuous variable is 0.8 (*μ*_*d *_= 1.517), the pair consistency probability for the dichotomized, trichotomized and quatrochotomized variables are 0.738, 0.775 and 0.787, respectively.

**Table 1:** Changes of the pair consistency probability *C *by the number of cutoff points

*μ*_d_*	C**	*C*_1_***	*C*_2_***	*C*_3_***
0.691	0.650	0.630	0.648	0.653
0.941	0.700	0.663	0.688	0.695
1.216	0.750	0.700	0.731	0.741
1.517	0.800	0.738	0.775	0.787
1.868	0.850	0.780	0.821	0.835
2.310	0.900	0.828	0.871	0.884
2.965	0.950	0.886	0.925	0.937

* Prognostic variables are assumed to satisfy *x*_[*h*] _~ *N*(0, 1^2^) and *x*_[*d*] _~ *N*(*μ*_*d*_, 1.5^2^)

** Pair consistency probability for the original continuous variable

*** Pair consistency probabilities for the categorized variables where suffixes indicate the number of cutoff points

Table 2 summarizes the means and standard deviations of the pair consistency probability *C *estimated by the parametric method for dichotomization when the sample sizes of the two groups are equal (*n *= 10, 25, 50, 100, 200, and 500) and *σ*_*d *_= 1.5*σ*_*h*_. Table 3 gives the results for trichotomization when the continuous variable in healthy and diseased cases has a common central tendency and the sample sizes of the two groups are equal. These tables can be used to evaluate the accuracy and precision of the estimated predictive ability of *C *for various sample sizes.

**Table 2:** Means and standard deviations of the estimates of *C *for a dichotomized variable

	true *C*		
*n*	0.650	0.750	0.850
10	0.662 ± 0.079	0.760 ± 0.076	0.856 ± 0.062
25	0.655 ± 0.051	0.754 ± 0.048	0.852 ± 0.040
50	0.652 ± 0.036	0.751 ± 0.034	0.851 ± 0.028
100	0.651 ± 0.026	0.751 ± 0.024	0.851 ± 0.020
200	0.650 ± 0.018	0.750 ± 0.017	0.850 ± 0.014
500	0.650 ± 0.011	0.750 ± 0.011	0.850 ± 0.009

Two samples of the size *n *from *N*(0, 1^2^) and *N*(*μ*_*d*_, 1.5^2^) are generated by Monte Carlo simulation and the pair consistency probability *C *for the optimal dichotomized point is calculated by the parametric method. This step is iterated 10,000 times, producing means and standard deviations of *C*. The values of *μ*_*d *_are 0.840, 1.613 and 2.543 for the true values of *C *0.650, 0.750 and 0.850, respectively.

**Table 3:** Means and standard deviations of the estimates of *C *for a trichotomized variable

	true *C*		
*n*	0.650	0.750	0.850
10	0.671 ± 0.065	0.759 ± 0.057	0.854 ± 0.039
25	0.658 ± 0.042	0.754 ± 0.036	0.851 ± 0.024
50	0.654 ± 0.030	0.752 ± 0.025	0.851 ± 0.017
100	0.652 ± 0.022	0.751 ± 0.017	0.850 ± 0.012
200	0.651 ± 0.015	0.751 ± 0.012	0.850 ± 0.008
500	0.651 ± 0.010	0.750 ± 0.008	0.850 ± 0.005

Two samples of the size *n* from *N*(0, 1^2^) and *N*(0, ) are generated by Monte Carlo simulation and the pair consistency probability *C* for the optimal trichotomized points is calculated by the parametric method. This step is iterated 10,000 times, producing means and standard deviations of *C*. The values of *σ_d_* are 1.898, 3.133 and 6.150 for the true values of *C* 0.650, 0.750 and 0.850, respectively.

### Example: Polychotomization of the prognostic factors of rhabdomyolysis

Rhabdomyolysis is a potentially lethal complication, often observed in patients who have attempted suicide with large doses of psychotropic drugs. Though it is important to make the diagnosis and begin proper treatment at an early stage, the diagnosis of rhabdomyolysis is difficult unless specific enzymes and myoglobin in skeletal muscle are detected by laboratory tests.

To find prognostic variables of rhabdomyolysis at an outpatient clinic where laboratory data are not available, we previously evaluated 131 cases of acute drug toxicosis [27-29] and found twelve variables to be significantly contributing to diagnosis of rhabdomyolysis (rhabdomyolysis group: *n *= 34, non-rhabdomyolysis group: *n *= 97). For this example, we selected three non laboratory data variables to predict the risk at the outpatient clinic: (1) *qtc: *ECG QTc (non-dimensional); (2) *t: *time from taking the drug to hospitalization (hours); and (3) *bt*, body temperature (Celsius).

Applying the maximum pair consistency probability criterion, the three continuous variables are categorized, assuming that *qtc *is a normal variable, *t *a log-normal variable and *bt *a variable with a common central tendency. Table 4 shows the selected cutoff points and the changes of the pair consistency probability. Comparing the pair consistency probabilities of the categorized variable, we can observe how predictive ability changes with polychotomization and the pair consistency probability *C *can be used as a measure to evaluate the loss of predictive ability by categorization.

**Table 4:** Optimal cutoff points for the prognostic factors of rhabdomyolysis

4a Optimal cutoff points forqtc*				
number of cutoff points	*z*_1_	*z*_2_	*z*_3_	*C*

1	0.460			0.611
2	0.428	0.491		0.634
3	0.410	0.460	0.509	0.642

continuous				0.651

4b Optimal cutoff points fort** *(hours)*				

number of cutoff points	*z*_1_	*z*_2_	*z*_3_	*C*

1	7.74			0.751
2	4.99	12.16		0.795
3	3.91	7.88	15.75	0.810

continuous				0.829

4c Optimal cutoff points forbt*** *(Celsius)*				

number of cutoff points	*z*_1_	*z*_2_		*C*

2	33.9	37.2		0.640

continuous				0.675

Abbreviations: *z*_1 _= first cutoff point; *z*_2 _= second cutoff point; *z*_3 _= third cutoff point

* Cutoff points for *qtc *(ECG QTc) are searched by the parametric method for normally distributed variables.

** Cutoff points for *t *(time from drug ingestion to arrival at hospital in hours) are searched assuming *t *is distributed log-normally.

*** Cutoff points for *bt *(body temperature in degrees Celsius) are searched by the parametric method for variables with a common central tendency.

Considering the predictive performance of the each of the categorized variables and convenience in the clinical setting, we finally chose the cutoff point values 0.45 for *qtc*, 5.0 and 12.0 for *t*, and 34.0 and 37.2 for *bt*. We then converted the continuous variables to categorical variables. Next, we applied the cross-split-half-method [30] to validate the effectiveness of prediction by these variables with logistic regression [31] and evaluated the amount of over estimation of prediction performance by a single data set. The estimated optimism for the overall *C *index was 0.018, which is sufficiently small.

### Example: Risk table for prognosis of rhabdomyolysis

Based on categorized variables, we obtained the new prediction formula:

*p *= 1/(1 + exp(7.96 - 3.13*QTC *- 6.22*T*_1 _- 3.11*T*_2 _- 1.97*BT*)     (8)

where *QTC *is ECG QTc (1 for more than or equal to 0.45 and 0 for less than 0.45), *T*_1 _is the time from drug ingestion to hospitalization (1 for more than or equal to 12 hours, 0 for otherwise), *T*_2 _is also the time from drug ingestion to hospitalization (1 for less than 12 hours and more than or equal to 5 hours, 0 for otherwise), and *BT *is body temperature (1 for more than or equal to 37.2° or less than or equal to 34.0°, and 0 for otherwise). Since the overall index *C *for this formula was 0.945, we estimate the predictive performance in future data will be around 0.927(= 0.945 - 0.018).

To ascertain the fitness of the selected regression model, we conducted the Hosmer-Lemeshow goodness-of-fit test [32] by dividing disease probability into eight classes. The actual number of occurrences for each class showed good agreement with the expected number of occurrences of rhabdomyolysis (p = 0.618).

Since all the three prognostic variables are categorized, the number of patient profiles becomes twelve and the risk probabilities of rhabdomyolysis for all possible patient profiles can now be obtained by assigning a combination of the values of categorized variables into regression formula (8). This yields a risk table for rhabdomyolysis occurrence (Table 5). For instance, if *T*, *QTC *and *BT *are "++ ", "+ " and "- " respectively, we can read from the table that the risk of rhabdomyolysis is 0.801. Repeated use of this table over time will give physicians a "sense" of the disease risk.

**Table 5:** Probability profile table for rhabdomyolysis

*T*	*QTC*	*BT*	*risk*
-	-	-	0.0003
-	-	+	0.0025
-	+	-	0.0079
-	+	+	0.0542
+	-	-	0.0078
+	-	+	0.0532
+	+	-	0.152
+	+	+	0.562
++	-	-	0.149
++	-	+	0.557
++	+	-	0.801
++	+	+	0.966

Abbreviations: *T *= time from taking the drug to arrival at hospital ('++' for more than or equal to 12 hours, '+' for less than 12 hours and more than or equal to 5 hours, '-' for less than 5 hours); *QTC *= ECG QTc ('+' for more than or equal to 0.45, '-' for less than 0.45); *BT *= body temperature ('+' for more than or equal to 37.2° or less than or equal to 34.0°, '-' for otherwise).

## Discussion

The criterion for optimal categorization of continuous variables in regression models may vary depending on the object of the categorization, and there have been several different approaches. Many of these approaches are inadequate for our purpose. We have proposed to use the overall discrimination index *C *introduced by Harrel and other authors [21-24] as the measure for predictive performance of a categorized variable. Since the overall discrimination index *C *has a clear and straight forward meaning as the pair consistency probability, it is intuitively logical to use it as a measure for the predictive discrimination for polychotomized variables.

Though mathematically distinct, our method has much in common with previously developed methods [2-20,33-38], which can be explained through the relations between the pair consistency probability *C*, SE and SP, and the area under the ROC straight line graph, as is expressed in formulae (2) to (6). In addition, our ROC straight line graph has a close relation with *ordinal dominance *or the OD curve proposed by Darlington to visualize the ordering feature of two comparative sets [39]. He showed that the OD curve is a complete representation of the rank-order properties of data and many statistical procedures follow naturally from assessment of the curve. Bamber clarified the relation between the area above the OD curve and a measure identical to the pair consistency probability [40]. Our proof of formula (6) related to the ROC straight line graph corresponds to Bamber's OD curve related proof.

Monte Carlo simulation showed that the naïve search of the maximum *C *index will give rise to an estimation bias, which is very much like the positive bias that affects the minimum *p*-value method. Such bias is also seen in the method where the cutoff point is selected in a way that maximizes the sum of SE and SP. Linnet and Brandt calculated the sample distribution of (SE + SP)/2 in the case of dichotomization using computer simulation assuming that distributions are normal, and evaluated the positive bias induced by the selection of an optimal cutoff point [4]. They found that estimates of test performance are too optimistic when the sample size is small, with an average positive bias up to 15% for a sample size of 25. We have shown that this problem does not affect our proposed parametric method.

However, there may be cases where a transformation to a normal distribution does not work well. For such cases, we conceive that approximation of distribution curve by a more suitable function or a restricted cubic spline function [41] creates a workable situation. We are currently in the process of evaluating this approach and the results will be reported elsewhere.

To keep this introduction of the maximum *C *index approach for polychotomizing predictive variables short and readable, we have used an example in which a regression model without correlated independent variables and without interaction fitted the observed data well (p = 0.618 by Hosmer and Lemeshow goodness-of-fit test) [42,43]. However, if correlation and interaction are relevant for the regression function, our maximum *C *index approach must be extended to a multivariable setting. Mazumdar extended a cutoff point search based on the maximum chi-square method to a multivariable setting [44], and showed that the cutoff points obtained by a multivariable search were closer to the true cutoff points.

Another method that is appealing for regression settings with correlated independent variables, is the so-called 'simplified integer score' method in which continuous variables are transformed into semi-continuous interval variables [41]. It has been used in numerous articles and is based on the categorization of the continuous variable, and the transformation of the products of the regression coefficient and the value of the variable into integers. This method is clinically useful and can be applied to the situation where explanatory variables are correlated. If the number of variables is small enough and they have few classifications, this method can also be used to create the simple probability profile tables that result from our approach. We are currently in the process of evaluating a multivariable extension of the *C *index maximization approach, including a comparison with this method.

Along with regression models, decision trees can also be used in diagnostic or prognostic decision making [36]. Breiman et al. developed an approach called *classification and regression trees *(CART) to build a decision tree for medical diagnosis based on a training data set [41,45]. In these decision trees, diagnosis is made by a sequential decision making process, in which a question on an independent variable is posed at each step and, depending on the answer, a different "branch" of the tree is selected until the final result is achieved. If an independent variable is continuous, dichotomization (or polychotomization) will be necessary to build a decision tree. Typically, the cutoff points are found by maximizing the total utility of decision scheme [46,47], which appears to be closely related mathematically to our approach. Further study is necessary to make a theoretical and practical comparison.

We have indicated that it is easier for most people to read a probability profile table to obtain the risk probability than to calculate the risk with a regression formula. Additionally, probability profile tables give physicians an intuitive feel for the disease risk. Even if the value of one or two of the prognostic variables is not available, physicians can obtain a probability range corresponding to the patient's risk by referring to both the positive and negative cases from the table. By making simplified risk tables in advance, physicians can obtain the patient's risk from an auxiliary table, even if the value of a predictor is missing. Since the table presentation of probabilities has these practical advantages, we believe our method for categorizing prognostic variables can be a helpful tool to make diagnostic or descriptive prognostic research with regression models become more applicable in clinical practice.

## Conclusion

We have proposed a new approach for polychotomization (including dichotomization) of independent continuous variables in regression models based on the overall discrimination index *C*, or the pair consistency probability, introduced by Harrel. We have shown that this index is closely related to the area under the ROC curve for the original continuous variable and that the resulting categorized variables have predictive properties comparable to the original continuous variable. We showed that the naïve application of the method gives rise to positive bias, not unlike the minimum *p*-value approach or the method of maximizing the sum of sensitivity and specificity, and we proposed a parametric version in which the estimates of the predictive performance and cutoff points are essentially unbiased. To evaluate the accuracy and precision of the estimate of the predictive performance, we presented tables of the means and standard deviations of the estimate of predictive performance for typical cases by the use of Monte Carlo simulation. Finally we provided an application of our method to a prediction rule with continuous predictor variables for rhabdomyolysis and showed that our method for polychotomizing continuous regressor variables can be a valid and useful tool to create probability profile tables. All programs (and their source codes) used in this study are available from the authors.

## Competing interests

The author(s) declare that they have no competing interests.

## Authors' contributions

HT derived the polychotomization method, drafted the manuscript and supervised the study. LB provided feedback on methodological issues and contributed to data analysis and manuscript writing. All authors read and approved the final manuscript.

## Pre-publication history

The pre-publication history for this paper can be accessed here:


